# Diverging diagnostic trajectories of degenerative cervical myelopathy and cervical radiculopathy in South Korea: a nationwide time-series analysis of claims data, 2010–2024

**DOI:** 10.3389/fpubh.2026.1948483

**Published:** 2026-10-01

**Authors:** Min Ki An, Je Il Ryu

**Affiliations:** 1 The Graduate School, Hanyang University, Seoul, Republic of Korea; 2 Department of Neurosurgery, College of Medicine, Hanyang University, Seoul, Republic of Korea

**Keywords:** aging, cervical vertebrae, epidemiology, insurance claim review, ossification of posterior longitudinal ligament, radiculopathy, Republic of Korea, spinal cord diseases

## Abstract

**Background:**

Degenerative cervical myelopathy (DCM) is the leading non-traumatic cause of spinal cord dysfunction in adults, and its burden is projected to grow as populations age. Cervical radiculopathy shares the same degenerative substrate but follows a separate diagnostic pathway. Long-term national trends in the two conditions, and their relation to surgical practice, are unquantified in an East Asian system with universal coverage.

**Methods:**

This nationwide repeated cross-sectional ecological analysis used aggregate open data from the Korean Health Insurance Review and Assessment Service, 2010 through 2024. DCM was identified by ICD-10 codes M50.0 and M47.1, cervical radiculopathy by M50.1, and ossification of the posterior longitudinal ligament by M48.8; fee codes identified surgical volumes. Rates are annual diagnostic prevalence (unique patients per code per year) and were age-standardized to the World Health Organization World Standard Population. Joinpoint regression quantified average annual percent change (AAPC), and four sensitivity analyses addressed case definition, care setting, coding era, and the March 2022 extension of magnetic resonance imaging reimbursement.

**Results:**

The DCM age-standardized rate fell from 147.2 to 83.7 per 100,000, a decline of 43.1 percent (AAPC −4.14 percent, 95 percent confidence interval [CI] −4.63 to −3.65, *p* < 0.001); the corresponding crude patient count fell 15.7 percent, whereas cervical radiculopathy rose from 697.9 to 851.9 (AAPC +1.34 percent, 95 percent CI +1.08 to +1.59). Total cervical procedure volume did not increase significantly (12,167 to 13,613; AAPC +0.60 percent, *p* = 0.078), but its composition shifted: posterior cervical fusion rose 105.5 percent and laminoplasty 120.8 percent, whereas anterior cervical discectomy and fusion changed little. The posterior share rose from 14.4 to 27.6 percent (AAPC +4.93 percent, 95 percent CI +4.53 to +5.34).

**Conclusion:**

The DCM age-standardized rate fell 43.1 percent over 15 years (crude patient count, 15.7 percent) while cervical radiculopathy rose, yet cervical surgical volume did not fall and laminoplasty more than doubled. The declining diagnosis rate is more consistent with a change in coding than with a decreasing burden of disease, and these estimates provide a baseline for surveillance of cervical spinal cord disease under universal coverage.

## 1 Introduction

Degenerative cervical myelopathy (DCM) is the leading non-traumatic cause of spinal cord dysfunction in adults (1). The neurological loss develops slowly. Once it is established, recovery is usually incomplete. With aging, disc degeneration, spondylosis, ligamentous hypertrophy, and ossification narrow the cervical canal, and the disease burden is therefore expected to increase in aging populations (2). In clinical practice DCM is handled as a spectrum overlapping other cervical spondylotic disorders (3, 4), and administrative studies have identified it with different codes and care settings, such as the hospital discharge code for cervical spondylosis with myelopathy in a Taiwanese national cohort (5). That variation complicates comparison of the population burden across periods and across countries. Long-term nationwide estimates of the diagnosed burden are needed for health-system planning, and for interpreting changes in surgical practice.

The anatomic substrate of cervical radiculopathy overlaps that of DCM. Natural history, treatment response, and age distribution separate the two conditions. A population-based study in Rochester, Minnesota reported a mean annual incidence of about 83 per 100,000 for cervical radiculopathy between 1976 and 1990, with the highest rates in the fifth and sixth decades (6). Western studies published later gave values in a similar range, but the case definitions they used were not the same, and this limits direct comparison (7). For Asian populations the available evidence is more limited. One Taiwanese national cohort followed cervical spondylotic myelopathy for many years (5). Korean nationwide work has dealt with lumbar radiculopathy, and no matched cervical myelopathy group was included in that analysis (8). The two conditions have therefore not been examined together in an Asian population.

South Korea offers a setting well suited to long-term epidemiologic surveillance of spinal disease. The country has operated a single-payer universal health coverage (UHC) scheme since 1989, with coverage approaching 97 percent of the resident population through the National Health Insurance Service (NHIS) and Medical Aid (9). Rapid population aging, policy-driven expansion of long-term care, and successive coverage reforms have made spinal degenerative disease a major component of the national disease burden (10). Prior Korean nationwide studies have characterized the overall healthcare burden of spinal disease (11, 12), reoperation risk after cervical surgery (13, 14), outcomes by surgical approach (15), lumbar surgical volume and cost-effectiveness (16), and the incidence of traumatic spinal cord injury (17). An earlier analysis of the same data source covered all spinal diagnoses (M40 to M54) in aggregate and did not separate cervical myelopathy from cervical radiculopathy (12). None of these studies directly compared long-term nationwide diagnosis trends between DCM and cervical radiculopathy, and none have explicitly addressed the possible confounding effect of the March 2022 expansion of National Health Insurance magnetic resonance imaging (MRI) reimbursement on observed diagnostic trends.

These trends were examined at the population level using aggregate claims data; individual-level clinical, radiological, or surgical outcomes are not within the scope of this design. Using Health Insurance Review and Assessment Service (HIRA) open data across 15 consecutive years, this study examined five aims: (1) to describe 15-year age-standardized rate (ASR) trajectories of annual diagnostic prevalence for DCM and cervical radiculopathy; (2) to quantify trend segments using joinpoint regression and to describe the 2020 pandemic year separately from the fitted models; (3) to describe demographic shifts in age and care setting within each disease group; (4) to describe cervical surgical procedure volumes and any shift between anterior and posterior approaches; and (5) to perform pre- and post-expansion stratified analyses addressing potential confounding by the National Health Insurance MRI reimbursement expansion.

## 2 Materials and methods

### 2.1 Study design and setting

The present analysis was designed as a nationwide, repeated cross-sectional, ecological time-series study of aggregate claims data spanning January 1, 2010 through December 31, 2024. Data were extracted from the HIRA public open-data portal, which publishes annual aggregate counts of primary-diagnosis claims, procedure claims, and expenditure for all insured services. Claims statements are filed as inpatient or outpatient; emergency department visits without admission are filed as outpatient, and rehabilitation care falls within the same two categories. The diagnosis release counts unique patients per calendar year for each code, once with all settings combined and once separately for inpatient and outpatient care, and the combined counts were used unless stated otherwise. HIRA data cover nearly all residents of the Republic of Korea through NHIS and Medical Aid, which together account for approximately 97 percent of the population under UHC (9, 18). Because only aggregate, fully de-identified, publicly released data were analyzed, the present analysis was exempt from review by an institutional review board under the relevant Korean bioethics regulations. The STROBE (Strengthening the Reporting of Observational Studies in Epidemiology) checklist for cross-sectional studies was used as the reporting framework. Counts of installed magnetic resonance imaging units were taken from the HIRA quarterly medical equipment statistics, available from 2018 onward.

### 2.2 Case definitions

The primary case definition for DCM combined ICD-10 codes M50.0 (cervical disc disorder with myelopathy) and M47.1 (other spondylosis with myelopathy). M47.1 is the ICD-10 category containing the successor of ICD-9-CM code 721.1, cervical spondylosis with myelopathy, which earlier national administrative studies used to identify the condition (5), and M50.0 covers disc-related cord compression. At the four-character level released by HIRA, M47.1 is not restricted to the cervical region, whereas M50.0 is cervical by definition; neither code has been validated against clinical records in Korean claims. Cervical radiculopathy was identified by M50.1, and ossification of the posterior longitudinal ligament (OPLL) by M48.8. Two alternative definitions were defined for each cohort. For DCM, the Narrow definition kept M50.0 alone. The Broad definition added G95.x (other diseases of the spinal cord) and G99.2 (myelopathy in diseases classified elsewhere) (19); these codes are not site-specific, so this definition is an upper bound rather than a cervical-specific count. The primary definition is the arithmetic sum of the M50.0 and M47.1 counts, so a patient carrying both codes in the same calendar year is counted twice. Because the Narrow, primary, and Broad definitions are nested, agreement among them is partly automatic; the two component codes were therefore also examined separately, which tests whether a change in dual coding could account for the primary-definition trend. For radiculopathy, the Narrow definition was restricted to M50.1 inpatient records, on the reasoning that severe radicular symptoms more often lead to admission.

All rates in this study are annual diagnostic prevalence: a patient is counted once per code in every calendar year with at least one claim carrying that code as the principal diagnosis. Individuals are not linked across years, so a patient with claims in several years is counted in each of those years. This quantity is referred to below as the diagnosis rate. A washout period cannot be applied to aggregate data, so the rates measure diagnostic activity rather than incidence.

Cervical surgical procedures were identified using the Korean fee codes listed in Ministry of Health and Welfare Notice No. 2025-13; fee codes of this kind have been used to identify cervical spine surgery in previous nationwide claims-based studies (13–15), although no validation against operative records has been published. These codes cannot be read at face value, because of the way billing is done in Korea. A cervical fusion is claimed under a principal fusion code, and the code that specifies the operative approach is entered as a secondary procedure. The secondary codes therefore indicate which approach was used, and they do not stand for separate operations. Five codes were used on this basis. N2463 is listed as cervical discectomy and indicates anterior cervical discectomy and fusion (ACDF). N0451 for cervical corpectomy is the principal code for anterior cervical corpectomy and fusion (ACCF). N2469 indicates posterior cervical fusion (PCF). N2491 is listed as cervical laminectomy and indicates cervical laminoplasty at the first level. N2492 is claimed once for every laminoplasty level after the first. Since N2492 is an add-on code and not a separate procedure, only N2463, N0451, N2469, and N2491 were counted as unique patient-level procedures. The procedure release is not cross-tabulated with diagnosis, so operations for trauma, infection, or neoplasm cannot be separated from degenerative cases, and primary and revision operations share the same codes; counts are unique patients per code per calendar year. The mean number of laminoplasty levels per patient was estimated as one plus the N2492 claim volume divided by the N2491 patient count, since N2491 is claimed once per patient and N2492 once per additional level. Claim N1493 for lumbar laminectomy was extracted as a contemporaneous non-cervical reference, in line with prior international work using administrative surgical fee codes for spine surgery analyses (20).

### 2.3 Statistical analysis

Annual denominators for rate calculation were obtained from the Korean Statistical Information Service mid-year resident population, released by sex and 5-year age group and summed across sex for the age-standardized denominators. Crude rates were calculated per 100,000 population. Direct age standardization was performed to the World Health Organization World Standard Population using 5-year age strata from 0–4 through 80 years and older. The WHO World Standard Population is the standard used in most international rate series and was therefore retained. This standard is younger than the Korean population, and crude counts and age-specific rates are therefore reported alongside the standardized rates.

Trend analysis used joinpoint regression with the National Cancer Institute Joinpoint Regression Program version 5.2. A log-linear model was fitted and the number of joinpoints was selected by grid search with the Monte Carlo permutation test (4,499 permutations, overall α = 0.05), following the methodology developed by Kim and colleagues (21, 22). All grid-search constraints were left at the program defaults, requiring a minimum of two observations between joinpoints and two observations from a joinpoint to either end of the series, with the maximum number of joinpoints set to two as recommended for a 15-point series. The annual percent change (APC) was estimated for each segment, and the average annual percent change (AAPC) was computed as a weighted mean of segment-specific APCs across the full study period. Trends were considered statistically significant at *p* < 0.05 when the 95 percent confidence interval (CI) excluded zero.

Four sensitivity analyses addressed potential sources of bias. Case-definition robustness was tested by applying three specifications (primary, Narrow, and Broad) to both the DCM and the radiculopathy cohorts. DCM trends were also re-estimated using inpatient records only. The purpose of this step was to check whether a change in outpatient coding could explain the main estimates. The Korean Classification of Diseases 7th revision (KCD-7) took effect on January 1, 2016, and estimates were stratified around that date to test for an administrative coding break. The reimbursement extension took effect on March 1, 2022 (Ministry of Health and Welfare Notice No. 2022-51); the pre-expansion window was therefore 2010–2021 and the post-expansion window 2023–2024, with 2022 dropped as a transition year. Surgical volume analyses were repeated under restriction to unique-procedure codes (11).

An annual series of 15 points is short, and this limitation affected several choices in the model. Deciding the number of change-points requires a sequence of tests, and the permutation procedure keeps the overall type I error rate under control across those tests. The multiple-comparison problem in segmented trend estimation was handled in this way. Setting an upper limit on the number of joinpoints also lowers the risk of overfitting when the series is short. The models were fitted with the uncorrelated-errors option of the program. Annual population rates may still be serially correlated, and if such dependence remains, the reported intervals become too narrow. Agreement among the three case definitions, and with the inpatient-restricted analysis, was used as an empirical check on the stability of the trends.

## 3 Results

### 3.1 Cohort characteristics

Between 2010 and 2024, the primary DCM patient count fell from 93,914 to 79,134 (−15.7 percent), cervical radiculopathy patients rose from 445,931 to 673,713 (+51.1 percent), and OPLL patients declined from 37,097 to 26,615 (−28.3 percent); the combined annual patient count rose from 576,942 to 779,462 (+35.1 percent) (Table 1). All counts are unique patients per calendar year rather than claim episodes. In 2024, the estimated mean age of the DCM group was 64.0 years (standard deviation [SD] 14.0) versus 56.3 years (SD 14.3) for the radiculopathy group, with 52.8 percent and 29.3 percent aged 65 years or older, respectively; the OPLL group had a mean age of 63.6 years (SD 14.4), with 50.8 percent aged 65 years or older. Within the M50.0 subgroup, the inpatient proportion nearly doubled from 6.1 percent to 11.7 percent.

**Table 1:** Demographic characteristics of the three diagnostic cohorts, South Korea, 2010 versus 2024.

Characteristic	DCM	Cervical radiculopathy	OPLL	Combined cohort^a^
	(M50.0 + M47.1)	(M50.1)	(M48.8)	
Patient count				
Patients in 2010, *n*	93,914	445,931	37,097	576,942
Patients in 2024, *n*	79,134	673,713	26,615	779,462
Change 2010 → 2024, %	−15.7	+51.1	−28.3	+35.1
Age at diagnosis, 2024^b^				
Mean (SD), years	64.0 (14.0)	56.3 (14.3)	63.6 (14.4)	—
≥65 years, %	52.8	29.3	50.8	—
Care setting (M50.0 subgroup)^c^				
Inpatient proportion 2010, %	6.1	—	—	—
Inpatient proportion 2024, %	11.7	—	—	—
Change, percentage points	+5.6	—	—	—

a Combined cohort is the arithmetic sum of the three diagnostic groups. The aggregate release counts patients within each ICD-10 group separately, so a patient carrying more than one of these diagnoses in the same calendar year is counted in more than one group; the combined figure is therefore an upper bound rather than a distinct patient count.

b Mean age and standard deviation are estimated from 5-year age bands using band midpoints; the open-ended band (80 years or older) was assigned a midpoint of 84 years. These estimates are therefore approximations constrained by the grouped structure of the aggregate data.

c Inpatient proportion is shown for the M50.0 subgroup and is calculated as inpatient patients divided by the sum of inpatient and outpatient patients.

### 3.2 Age-standardized rates and joinpoint regression

The DCM ASR per 100,000 fell from 147.2 in 2010 to 83.7 in 2024. The cervical radiculopathy ASR rose from 697.9 to 851.9, and the OPLL ASR fell from 57.9 to 28.5 (Table 2). No joinpoint reached statistical significance for DCM, whose rate is therefore described by a single log-linear trend across the fifteen years (AAPC −4.14 percent, 95 percent CI −4.63 to −3.65, *p* < 0.001) (Table 3; Figure 1).

**Table 2:** Year-by-year age-standardized diagnosis rates per 100,000, South Korea, 2010–2024.

Year	DCM ASR	(95% CI)	Radiculopathy ASR	(95% CI)	OPLL ASR	(95% CI)
2010	147.2	(146.3–148.2)	697.9	(695.8–700.0)	57.9	(57.3–58.5)
2011	148.4	(147.5–149.4)	770.8	(768.6–772.9)	58.8	(58.2–59.4)
2012	144.9	(143.9–145.8)	825.2	(823.0–827.4)	59.0	(58.4–59.6)
2013	134.3	(133.5–135.2)	825.3	(823.1–827.5)	60.2	(59.6–60.8)
2014	133.6	(132.8–134.5)	850.2	(848.0–852.4)	60.5	(59.9–61.0)
2015	125.4	(124.6–126.2)	838.8	(836.6–841.0)	60.1	(59.6–60.7)
2016	124.4	(123.6–125.2)	862.4	(860.1–864.6)	55.9	(55.4–56.5)
2017	118.7	(117.9–119.5)	887.6	(885.3–889.8)	50.4	(49.9–51.0)
2018	121.0	(120.2–121.8)	901.2	(899.0–903.5)	48.4	(47.9–48.9)
2019	111.9	(111.2–112.7)	932.6	(930.4–934.9)	42.5	(42.1–43.0)
2020	97.1	(96.4–97.8)	883.0	(880.8–885.3)	35.1	(34.7–35.5)
2021	94.3	(93.6–95.0)	888.5	(886.3–890.8)	35.4	(35.0–35.9)
2022	92.4	(91.7–93.1)	861.8	(859.6–864.0)	33.1	(32.7–33.5)
2023	89.9	(89.2–90.5)	862.9	(860.7–865.2)	30.8	(30.4–31.1)
2024	83.7	(83.0–84.3)	851.9	(849.7–854.2)	28.5	(28.1–28.9)

Rates are age-standardized to the World Health Organization World Standard Population using 5-year age strata (0–4, 5–9, …, 75–79, and 80 years or older). Denominators are Korean mid-year (annual average) registered resident populations from the Korean Statistical Information Service. Confidence intervals are computed under the Poisson distribution.

**Table 3:** Joinpoint regression of age-standardized diagnosis rates, South Korea, 2010–2024.

Disease group	Years	APC or AAPC, % (95% CI)	*p*	Joinpoints
Degenerative cervical myelopathy				None
Full period (AAPC)	2010–2024	−4.14 (−4.63 to −3.65)	<0.001	
Cervical radiculopathy				2012, 2019
Segment	2010–2012	+7.88 (+6.01 to +9.78)	<0.001	
Segment	2012–2019	+1.55 (+1.13 to +1.97)	<0.001	
Segment	2019–2024	−1.46 (−2.06 to −0.86)	<0.001	
Full period (AAPC)	2010–2024	+1.34 (+1.08 to +1.59)	<0.001	
Ossification of the posterior longitudinal ligament				2015
Segment	2010–2015	+0.65 (−0.85 to +2.17)	0.366	
Segment	2015–2024	−8.29 (−8.99 to −7.58)	<0.001	
Full period (AAPC)	2010–2024	−5.19 (−5.64 to −4.74)	<0.001	

APC = annual percent change; AAPC = average annual percent change; CI = confidence interval. Rates are age-standardized to the World Health Organization World Standard Population. Joinpoints were selected by grid search with the Monte Carlo permutation test (4,499 permutations, overall α = 0.05) under the program’s default constraints, which require a minimum of two observations between joinpoints and two from a joinpoint to either end of the series. No joinpoint reached statistical significance for degenerative cervical myelopathy, which is therefore described by a single log-linear trend. For that group the AAPC is therefore identical to the single segment APC.

**Figure 1:**
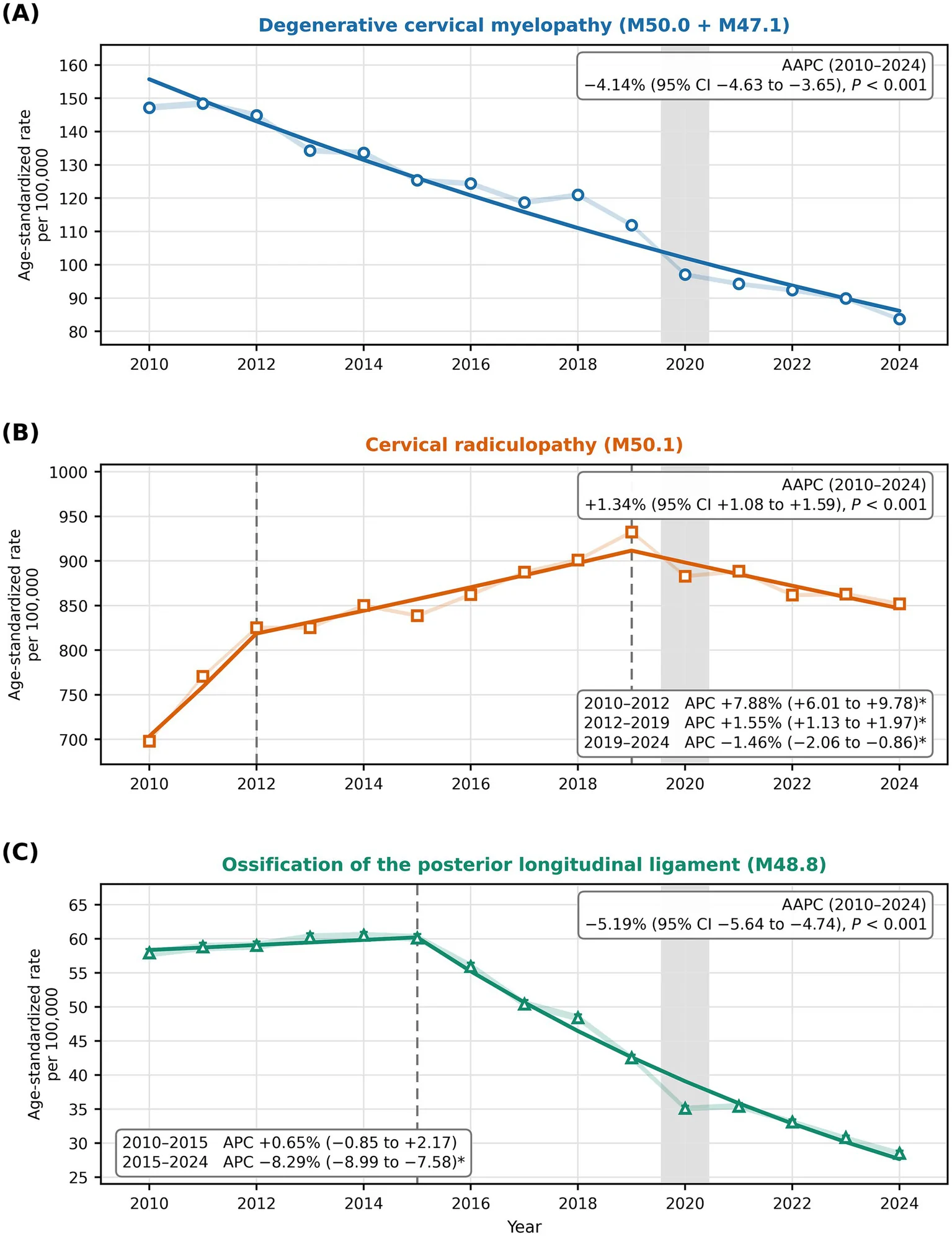
Age-standardized diagnosis rate trends, 2010–2024. **(A)** Degenerative cervical myelopathy. **(B)** Cervical radiculopathy. **(C)** Ossification of the posterior longitudinal ligament. Solid lines show the fitted joinpoint model; markers show observed annual rates per 100,000; shaded ribbons and error bars indicate 95 percent confidence intervals. Dashed vertical lines mark joinpoint locations; no joinpoint reached significance for degenerative cervical myelopathy, which is shown as a single fitted segment. The grey vertical band identifies calendar year 2020 (COVID-19 pandemic). Boxes within each panel report segment-specific annual percent change (APC) with 95 percent confidence intervals and the average annual percent change (AAPC) for 2010–2024. Asterisks indicate *p* < 0.05.

For cervical radiculopathy, two joinpoints were identified, at 2012 and 2019, and the AAPC was +1.34 percent (95 percent CI +1.08 to +1.59, *p* < 0.001). Segment APCs were +7.88 percent for 2010–2012 (95 percent CI +6.01 to +9.78, *p* < 0.001), +1.55 percent for 2012–2019 (95 percent CI +1.13 to +1.97, *p* < 0.001), and −1.46 percent for 2019–2024 (95 percent CI −2.06 to −0.86, *p* < 0.001), so the rise reversed after 2019. OPLL showed a single joinpoint at 2015, with an AAPC of −5.19 percent (95 percent CI −5.64 to −4.74, *p* < 0.001); the rate did not change significantly before 2015 (APC +0.65 percent, 95 percent CI −0.85 to +2.17, *p* = 0.366) and fell thereafter (APC −8.29 percent, 95 percent CI −8.99 to −7.58, *p* < 0.001) (Table 3).

Summed across the two conditions, the combined DCM-radiculopathy age-standardized rate rose from 845.1 to 935.6 per 100,000, an increase of 10.7 percent (AAPC +0.63 percent, 95 percent CI +0.36 to +0.90). The combined burden captured by these two labels therefore increased modestly while its internal composition shifted.

The pandemic year is described separately, without reference to the fitted models. Between 2019 and 2020 the DCM rate fell 13.2 percent, against a mean annual change of −2.81 percent over the preceding four years; the cervical radiculopathy rate fell 5.3 percent, reversing a mean annual rise of +2.69 percent; and the OPLL rate fell 17.4 percent. Rates in 2021 stayed close to their 2020 values (DCM −2.9 percent, cervical radiculopathy +0.6 percent, OPLL +0.9 percent), so the 2020 disruption was not followed by a rebound within the observation window.

Both rates declined after 2019. Calculated from the observed endpoint rates rather than from the fitted models, the DCM rate fell at 5.65 percent per year and the cervical radiculopathy rate at 1.79 percent per year between 2019 and 2024. The two series therefore continued to separate over this period, by 3.86 percentage points per year, although less steeply than the 6.27 percentage points per year that separated them between 2010 and 2019.

### 3.3 Cervical surgical procedure volumes

Total unique cervical procedures rose 11.9 percent over the study period, from 12,167 in 2010 to 13,613 in 2024 (Table 4). The composition of that total changed more than the total itself. ACDF (N2463) remained close to its 2010 level, at 9,692 and 9,557 in 2024, a decline of 1.4 percent, while cervical corpectomy (N0451), the primary code for ACCF, decreased from 722 to 293, or 59.4 percent. Posterior codes changed in the opposite direction. PCF (N2469) increased from 705 to 1,449, a gain of 105.5 percent, and first-level cervical laminoplasty (N2491) from 1,048 to 2,314, a gain of 120.8 percent. Adding the N2492 claim volume to the first level implied a mean of approximately 2.76 laminoplasty levels per patient in 2024. The posterior approach share, calculated as (N2469 + N2491) divided by total unique procedures, rose from 14.4 percent to 27.6 percent, a gain of 13.2 percentage points. Fitting the full annual series, rather than taking the difference between two endpoint years, confirmed a continuous rise: AAPC +4.93 percent (95 percent CI +4.53 to +5.34, *p* < 0.001). Posterior volume increased as well, at an AAPC of +5.38 percent (95 percent CI +4.63 to +6.13, *p* < 0.001). Anterior volume did not. Its AAPC was −0.67 percent (95 percent CI −1.39 to +0.05, *p* = 0.066), and total cervical procedure volume was likewise stable at +0.60 percent (95 percent CI −0.08 to +1.29, *p* = 0.078).

**Table 4:** Cervical surgical procedure volumes and annual trends, South Korea, 2010–2024.

Procedure (fee code)	2010, *n*	2024, *n*	Change, %	AAPC, % (95% CI)	*p*
Anterior approach					
ACDF (N2463)	9,692	9,557	−1.4	−0.39 (−1.14 to +0.36)	0.276
ACCF (N0451)	722	293	−59.4	−6.42 (−7.39 to −5.43)	<0.001
Subtotal	10,414	9,850	−5.4	−0.67 (−1.39 to +0.05)	0.066
Posterior approach					
PCF (N2469)	705	1,449	+105.5	+5.18 (+4.25 to +6.11)	<0.001
Laminoplasty (N2491)	1,048	2,314	+120.8	+5.83 (+5.05 to +6.61)	<0.001
Subtotal	1,753	3,763	+114.7	+5.38 (+4.63 to +6.13)	<0.001
Overall					
Total unique procedures	12,167	13,613	+11.9	+0.60 (−0.08 to +1.29)	0.078
Posterior approach share, %	14.4	27.6	+13.2 pp	+4.93 (+4.53 to +5.34)	<0.001
Non-cervical reference					
Lumbar laminectomy (N1493)	80,720	65,926	−18.3	−1.30 (−1.80 to −0.80)	<0.001

ACDF = anterior cervical discectomy and fusion; ACCF = anterior cervical corpectomy and fusion; PCF = posterior cervical fusion; AAPC = average annual percent change; pp = percentage points. Counts are unique patients per procedure code per calendar year. N2492 (each additional laminoplasty level) is an add-on marker and is therefore excluded from unique-procedure counts. AAPC values are derived from joinpoint regression of the full 15-point annual series (2010–2024), not from the two endpoint years. Change, % compares the two endpoint years, whereas AAPC is fitted to the full 15-point annual series, so the two need not agree in magnitude.

### 3.4 Sensitivity analyses

The Narrow and primary definitions produced similar estimates (Narrow −3.63 percent, primary −4.14 percent), whereas the Broad definition declined more slowly (−2.66 percent, 95 percent CI −3.01 to −2.30) because the site-nonspecific codes it adds rose over the same period (Table 5). Since these definitions are nested, the two component codes of the primary definition were examined on their own: M50.0 alone fell from 65.0 to 38.3 per 100,000 and M47.1 alone from 82.2 to 45.4, at AAPCs of −3.63 percent (95 percent CI −3.98 to −3.29) and −4.24 percent (95 percent CI −4.99 to −3.47), so the decline is not attributable to either code alone. Each component declined independently, and the primary-definition AAPC of −4.14 percent lies between the two; a change in dual coding therefore cannot account for the primary-definition trend. Restricting the DCM cohort to inpatient records reversed the direction of the trend: inpatient-only counts rose from 4,164 to 7,744 (AAPC +4.20 percent, 95 percent CI +3.29 to +5.13, *p* < 0.001). That series is a crude count, because the aggregate release does not cross-tabulate care setting with age, and population aging over the same period contributes to the increase. The inpatient proportion within M50.0 carries the same age structure in numerator and denominator and is less exposed to that effect; it rose from 6.1 percent to 11.7 percent. Era-stratified estimates showed a decline in both coding eras (2010–2015, AAPC −3.35 percent, 95 percent CI −4.87 to −1.80, *p* = 0.004; 2016–2024, AAPC −5.09 percent, 95 percent CI −6.17 to −3.99, *p* < 0.001), so the fall does not reflect the KCD-7 transition alone.

**Table 5:** Sensitivity analyses: case definitions, care setting, and coding era, 2010–2024.

Case definition	ICD-10 codes	2024, n	2010 → 2024^a^	AAPC, % (95% CI)	*p*
Degenerative cervical myelopathy					
Narrow	M50.0 only	33,960	65.0 → 38.3	−3.63 (−3.98 to −3.29)	<0.001
M47.1 alone	M47.1 only	45,174	82.2 → 45.4	−4.24 (−4.99 to −3.47)	<0.001
**Primary (base)**	M50.0 + M47.1	**79,134**	**147.2 → 83.7**	−4.14 (−4.63 to −3.65)	<0.001
Broad	M50.0 + M47.1 + G95.x + G99.2	105,073	166.1 → 114.4	−2.66 (−3.01 to −2.30)	<0.001
Cervical radiculopathy					
Narrow a	M50.1, inpatient only	45,822	20,562 → 45,822	+5.20 (+4.31 to +6.10)	<0.001
**Primary (base)**	M50.1	**673,713**	**697.9 → 851.9**	+1.34 (+1.08 to +1.59)	<0.001
Broad	M50.1 + M50.2 + M50.3 + M50.8 + M50.9	1,012,035	1129.8 → 1288.2	+0.91 (+0.58 to +1.24)	<0.001
Care-setting restriction^a^					
Inpatient-only DCM	M50.0 + M47.1, inpatient	7,744	4,164 → 7,744	+4.20 (+3.29 to +5.13)	<0.001
Coding-era stratification^b^					
KCD-6 era (2010–2015)	M50.0 + M47.1	—	2010 → 2015	−3.35 (−4.87 to −1.80)	0.004
KCD-7 era (2016–2024)	M50.0 + M47.1	—	2016 → 2024	−5.09 (−6.17 to −3.99)	<0.001
OPLL, KCD-6 era	M48.8	—	2010 → 2015	+0.84 (+0.35 to +1.33)	0.009
OPLL, KCD-7 era	M48.8	—	2016 → 2024	−8.19 (−9.46 to −6.90)	<0.001

ASR = age-standardized rate per 100,000; AAPC = average annual percent change. All estimates use the same joinpoint specification as the main analysis (maximum two joinpoints, Monte Carlo permutation test with 4,499 replicates, program default spacing constraints), except that the era-stratified rows contain too few annual observations to admit two joinpoints; those rows were fitted with the maximum permitted by the series length, which was none for 2010–2015 and one for 2016–2024. Convergence of the three myelopathy definitions within a narrow AAPC range indicates that the observed decline is not an artifact of a single code selection.

a Rows marked a report crude patient counts rather than age-standardized rates and are not directly comparable with the rate-based rows. The inpatient-only analysis tests whether a shift in outpatient coding accounts for the overall myelopathy decline. Because the aggregate release does not stratify care setting by age, this row reports crude patient counts rather than age-standardized rates and is therefore not directly comparable with the rate-based rows above; population aging over the same period contributes to the observed rise. The near-doubling of the M50.0 inpatient share shown in Table 1 carries the same age structure in numerator and denominator, is less exposed to that effect, and is consistent with progressive case-mix severity within a contracting diagnostic label.

b The Korean Classification of Diseases 7th revision (KCD-7) took effect on January 1, 2016. Era-stratified estimates test whether an observed trend reflects an administrative coding break rather than a continuing change. For DCM the decline is present in both eras. For OPLL the direction reverses at the era boundary, from a rise before 2016 to a fall thereafter, which is the pattern expected when a classification change alters coding. Values in bold indicate the primary (base) case definition used in the main analysis.

For cervical radiculopathy, the Broad definition (M50.1 + M50.2 + M50.3 + M50.8 + M50.9) yielded an AAPC of +0.91 percent (95 percent CI +0.58 to +1.24) with 1,012,035 patients in 2024, and the Narrow definition, restricted to inpatient records, rose from 20,562 to 45,822 patients (AAPC +5.20 percent, 95 percent CI +4.31 to +6.10). Inpatient counts thus increased for radiculopathy as well as for DCM, and the inpatient increase is not limited to the myelopathy label. The inpatient proportion is less affected by population aging, and this proportion rose more steeply within M50.0 than within M50.1, where it moved from 4.5 to 6.5 percent.

### 3.5 Pre- and post-expansion stratified analysis

The reimbursement extension took effect on March 1, 2022, so twelve of the fifteen observation years precede the policy. Across that pre-expansion window (2010–2021), the DCM ASR fell steadily (AAPC −4.24 percent, 95 percent CI −4.74 to −3.73, *p* < 0.001) while the cervical radiculopathy ASR rose (AAPC +2.42 percent, 95 percent CI +1.93 to +2.91, *p* < 0.001), and the posterior approach share climbed from 14.4 to 25.8 percent (AAPC +5.57 percent, 95 percent CI +4.95 to +6.19, *p* < 0.001) (Table 6; Supplementary Figure S1). The post-expansion window contains only two annual observations after exclusion of the 2022 transition year, which is insufficient for regression-based trend estimation; DCM fell from 89.9 to 83.7 and radiculopathy from 862.9 to 851.9 across those two years, with no reversal of the preceding trajectories.

**Table 6:** Stratified analysis around the March 2022 spinal MRI reimbursement extension.

Outcome	Period	Start	End	AAPC, % (95% CI)	*p*
DCM ASR	Pre-expansion (2010–2021)	147.2	94.3	−4.24 (−4.74 to −3.73)	<0.001
	Post-expansion (2023–2024)	89.9	83.7	Not estimable^a^	—
Cervical radiculopathy ASR	Pre-expansion (2010–2021)	697.9	888.5	+2.42 (+1.93 to +2.91)	<0.001
	Post-expansion (2023–2024)	862.9	851.9	Not estimable^a^	—
OPLL ASR	Pre-expansion (2010–2021)	57.9	35.4	−4.61 (−5.21 to −4.00)	<0.001
	Post-expansion (2023–2024)	30.8	28.5	Not estimable^a^	—
Posterior approach share, %	Pre-expansion (2010–2021)	14.4	25.8	+5.57 (+4.95 to +6.19)	<0.001
	Post-expansion (2023–2024)	27.7	27.6	Not estimable^a^	—

a The 2022 transition year is excluded because the reimbursement extension took effect on March 1, 2022. The post-expansion window contains only two annual observations, which is insufficient for regression-based trend estimation; a regression-based interrupted time-series model would be over-fitted and underpowered in this window. Twelve of the fifteen observation years therefore precede the policy.

The direction of both trends held across age. Between 2010 and 2024 the DCM rate fell in every age band examined, from 36.7 to 21.8 per 100,000 under 40 years, from 203.5 to 117.6 at 40 to 54, from 408.8 to 221.2 at 55 to 64, from 721.4 to 378.1 at 65 to 74, and from 689.5 to 478.4 at 75 years or older, at annual rates between −2.82 and −4.76 percent. Cervical radiculopathy rose in four of the five bands and showed no significant change at 55 to 64 (AAPC +0.24 percent, 95 percent CI −0.09 to +0.58). The steepest proportional rises were under 40 years (AAPC +3.41 percent) and at 75 years or older (+3.05 percent), but the two bands differ greatly in absolute terms: 85 percent of the increase in radiculopathy patients occurred at 55 years or older and 7 percent under 40. At 65 to 74 the fall in the DCM rate and the rise in the radiculopathy rate were of almost equal size, −343.3 and +365.0 per 100,000 (Table 7).

**Table 7:** Age-specific diagnosis rates per 100,000 population, South Korea, 2010–2024.

Age group	DCM 2010 → 2024	DCM change	DCM AAPC, % (95% CI)	Radiculopathy 2010 → 2024	Radiculopathy change	Radiculopathy AAPC, % (95% CI)
Under 40	36.7 → 21.8	−14.9	−3.87 (−4.40 to −3.35)	258.3 → 419.2	+160.9	+3.41 (+3.10 to +3.72)
40–54	203.5 → 117.6	−85.9	−4.17 (−4.69 to −3.65)	1496.3 → 1697.9	+201.6	+0.78 (+0.51 to +1.06)
55–64	408.8 → 221.2	−187.7	−4.52 (−5.00 to −4.04)	2072.6 → 2182.0	+109.3	+0.24 (−0.09 to +0.58)
65–74	721.4 → 378.1	−343.3	−4.76 (−5.29 to −4.24)	1890.1 → 2255.1	+365.0	+1.15 (+0.77 to +1.53)
75 or older	689.5 → 478.4	−211.1	−2.82 (−3.44 to −2.18)	993.3 → 1606.0	+612.7	+3.05 (+2.65 to +3.45)

DCM = degenerative cervical myelopathy; AAPC = average annual percent change; CI = confidence interval. Rates are patients per 100,000 population within each age band, calculated with mid-year resident population denominators for the same band; they are age-specific and are therefore not standardized. Change is the difference in rate between 2010 and 2024. Changes are computed from unrounded rates and may therefore differ by 0.1 from the difference of the rounded values shown. AAPC values are from joinpoint regression of the full 15-point annual series under the program’s default constraints. The radiculopathy AAPC at 55 to 64 was not statistically significant; all other estimates had *p* < 0.001.

## 4 Discussion

The central finding is a contrast between two indicators drawn from the same claims system. The age-standardized diagnosis rate for DCM fell by 43.1 percent while total cervical surgical volume did not decline (AAPC +0.60 percent, *p* = 0.078) and laminoplasty, a posterior decompression used for multi-level disease (13, 15), more than doubled. Cervical surgery for myelopathy is indicated by progressive neurological deficit (4), so a real fall in the number of patients with myelopathy would be expected to reduce the number who reach the threshold for surgery; the operative record shows no such reduction. This contrast is the principal reason to interpret the diagnostic decline as a change in recording rather than in disease occurrence, and the mechanisms considered below are therefore assessed against it.

The observed DCM decline and radiculopathy rise may reflect more than one mechanism at the same time. Broader imaging access is one factor. Installed magnetic resonance imaging capacity in Korea rose by about a third between 2018 and 2024, from 1,553 to 2,035 units. Higher rates of advanced spinal imaging have been linked to higher rates of spine surgery (23); reducing out-of-pocket payment raises imaging use across income groups in Korea (24); and a substantial share of cervical spine studies performed under such conditions returns negative findings (25). Patients who would previously have been coded M50.0 or M47.1 may now be coded M50.1 instead. The two changes are mismatched in size. M50.1 rose 51.1 percent. DCM fell 15.7 percent. Simple code redistribution between these two categories therefore explains only a small portion of the radiculopathy rise. Most of it likely reflects private supplementary insurance, population aging, and expanded outpatient care for mild cervical symptoms.

The shape of the DCM series constrains how these mechanisms can be invoked. No joinpoint reached significance across the fifteen years, so the decline is a single uninterrupted log-linear trend rather than a step tied to any dated event. If one event, such as a reimbursement change or a sudden increase in imaging capacity, had driven the decline, the slope should have changed in the year of that event. No such change reached statistical significance, although a 15-point annual series has limited power to detect a modest change in slope. The observed pattern is more consistent with a slow change in diagnostic and coding practice that accumulated over more than ten years, and not with a reaction to one policy or one technology. Wider access to imaging is therefore treated here as part of that slow background, and not as a cause with a specific date.

Two features of the data suggest that the decline is not produced by age standardization. The WHO World Standard Population is younger than the Korean population. Standardization therefore makes a fall appear larger when the condition occurs mainly at older ages, and the crude patient count fell 15.7 percent while the age-standardized fall was 43.1 percent. The age-specific rates in Table 7 are not standardized at all, and they fell in every band examined, at annual rates between −2.82 and −4.76 percent. The direction of the decline, and its approximate size, therefore do not depend on which standard population is chosen. Whether one code is responsible for the fall is a separate question. The two codes of the primary definition declined independently of each other, M50.0 at an AAPC of −3.63 percent and M47.1 at −4.24 percent (4, 13, 26). The Broad definition adds myelopathy codes that are not site-specific, and it declined more slowly, because those codes increased while the two cervical-specific codes decreased. Taken together, these patterns are more consistent with a move toward less specific labels than with a fall in disease occurrence.

These trends fit the Korean demographic and health-system context. Korea is aging faster than almost any other Organisation for Economic Co-operation and Development country, and UHC expansion during the study period reduced financial barriers to outpatient care (9, 10). Long-term East Asian data are limited. A Taiwanese national cohort reported a rising cervical spondylotic myelopathy rate in the late 2000s (5). The DCM decline observed here therefore appears to reflect a health system with rapid MRI expansion under UHC rather than a universal epidemiologic pattern. The radiculopathy rise is consistent with other reports (6, 7), but the 51.1 percent Korean increase is near the upper end of international estimates. NHIS coverage expansion, growth of private supplementary insurance, and increased outpatient utilization for cervical degenerative disease likely account for this (11, 27).

The absolute level requires separate comment. The radiculopathy rate reported here is roughly ten times the Rochester estimate (6), and the two numbers measure different quantities. The Rochester cohort counted incident cases, which were identified by medical record review within a defined observation window. The counts reported here are an annual diagnostic prevalence. Any patient with an M50.1 claim during a calendar year is included in the numerator, whether the diagnosis was first made in that year or ten years before. A health system in which imaging is inexpensive and outpatient access is not restricted will also record the same patient more often. These rates therefore reflect diagnostic activity in one health system over time, and not the rate at which the disease arises. Comparison of their absolute level between countries is not meaningful.

The shift toward posterior surgical approaches requires separate discussion. Over the fifteen years the composition of the cervical surgical workload changed, whereas its size did not. ACDF volume changed little, and N0451 (ACCF) fell by more than half. Korean reoperation studies have reported different revision risk profiles for ACDF, PCF, and laminoplasty, and laminoplasty has become more widely used as a motion-preserving posterior option in multi-level disease (14, 15, 28). Lumbar laminectomy (N1493) fell by 18.3 percent over the same years, so the cervical shift toward posterior surgery appears specific to cervical practice rather than part of a general expansion of spine surgery (16, 29).

The OPLL result requires further explanation. M48.8 coding fell at an AAPC of −5.19 percent, yet cervical laminoplasty volume more than doubled across the same years. Korean claims are aggregated by principal diagnosis, and a patient who carries OPLL together with cord compression is usually admitted under M50.0 or M47.1, with M48.8 entered as a secondary code. Tables built on the principal diagnosis will therefore under-count the operative burden of OPLL, and the fall in M48.8 coding is unlikely to mean that OPLL itself is becoming less common (13). The same reasoning raises a question about the DCM series, since aggregation that obscures OPLL might in principle also generate an apparent myelopathy decline. Two observations indicate that the DCM series is not subject to the same effect. M50.0 and M47.1 are the labels under which such patients are admitted, so cases are added to rather than removed from these codes when coding is assigned by principal diagnosis at admission. The inpatient proportion within M50.0 rose from 6.1 to 11.7 percent while the overall rate fell, which is the opposite of what displacement out of the myelopathy label at the inpatient end would produce. The decline is therefore concentrated in the outpatient setting, where the diagnostic threshold is least constrained.

The era-stratified analysis supports the view that the myelopathy decline is not an artifact of a classification change, and it also shows that the test itself has power to detect such a change. Applying the same stratification around the Korean Classification of Diseases 7th revision, which took effect on January 1, 2016, the OPLL rate rose before the boundary (APC +0.84 percent, 95 percent CI +0.35 to +1.33, *p* = 0.009) and fell steeply afterwards (−8.19 percent, 95 percent CI −9.46 to −6.90). The joinpoint fitted to the full OPLL series was located at 2015, adjacent to the January 2016 boundary and within the resolution that 15 annual observations allow. The corresponding segment in that model (APC +0.65 percent, 95 percent CI −0.85 to +2.17) is estimated with a wider interval because it also carries the uncertainty in the position of the joinpoint, whereas the era-stratified estimate fits a fixed, externally defined boundary. A reversal of direction at a classification boundary is the pattern expected when a classification change alters coding. The same test applied to DCM found a decline in both eras (−3.35 and −5.09 percent), with no reversal. OPLL therefore serves as a positive control: the stratification detects a coding break where one is plausible, and does not detect one in the myelopathy series.

Diagnostic trajectories diverged while surgical practice moved toward posterior decompression, but these two observations come from different levels of the claims system. Coding myelopathy in an outpatient setting requires a neurological examination and correlation with imaging, whereas radicular symptoms can be coded from the presenting complaint. Mild myelopathic signs may go undetected in a brief consultation, and an international analysis has shown that ICD-10 coding of cervical radiculopathy under-captures concurrent myelopathy (19). A mild myelopathic case coded as M50.1 would raise the radiculopathy count and lower the DCM count at the same time. Planning for multi-level surgery depends on a different set of factors, namely imaging, sagittal alignment, and symptom progression, and not on outpatient coding practice. These two interpretations are not mutually exclusive. Migration of milder cases toward M50.1 in the outpatient setting would itself concentrate severity within the remaining M50.0 label. The near-doubling of the M50.0 inpatient share described above is consistent with such a concentration.

The pre- and post-expansion stratification addresses the most plausible policy confounder directly. On March 1, 2022 the National Health Insurance extended spinal MRI reimbursement to degenerative disease severe enough to warrant surgery. The design of that policy limits how far it can account for the DCM series. Spinal cord disease was already a reimbursed indication before the extension, and what the 2022 change added was coverage for degenerative disease without myelopathy. Earlier detection of cervical cord compression could in principle either raise DCM counts through better ascertainment or lower them through earlier treatment before the counted DCM phase, but the extension is more directly relevant to the radiculopathy end of the spectrum than to DCM. Across the twelve pre-expansion years the DCM rate fell at −4.24 percent per year while the radiculopathy rate rose at +2.42 percent per year, so the divergence between the two conditions was fully established before the reimbursement change. Rates fell sharply in all three groups in 2020 without a corresponding joinpoint in any of the fitted models. The fall was confined to a single year and was not followed by a rebound in 2021, which is consistent with a short-term disruption of outpatient access reported internationally and in Korean spinal series (30–32).

### 4.1 Limitations

Claims data reflect physician billing and coding behavior rather than the underlying anatomic or pathologic state of patients, and that caveat frames every interpretation offered here. No published study has validated cervical myelopathy or radiculopathy coding in Korean claims; three case definitions were applied as sensitivity analyses to address this in part, following Korean validation work showing that diagnosis codes combined with procedure or prescription claims perform better than diagnosis codes alone (33, 34). Coding drift between M50.0, M47.1, and M48.8 remains likely (19). M48.8 covers other specified spondylopathies and is not exclusive to ossification of the posterior longitudinal ligament, so that cohort is broader than its label suggests. The mapping of fee codes to surgical approach rests on billing convention rather than on a published validation study, and a secondary procedure claim cannot be verified against an operative record. Procedure counts are not cross-tabulated with diagnosis, so operations for trauma, infection, or neoplasm are included, and revision operations in later years are counted again; the non-degenerative fraction cannot be quantified from the aggregate release. The posterior-approach share would be distorted only if that fraction differed by approach and changed over time.

The ecological design does not allow individual-level causal inference. Because the open data are aggregated, comorbidity, income, secondary diagnoses, and overlap between OPLL and DCM cannot be adjusted for, and the counts reflect annual diagnostic prevalence rather than true incidence, since a washout period cannot be applied. Care setting is not cross-tabulated with age, so the inpatient-restricted analysis rests on crude counts and the contribution of population aging cannot be separated from a change in case mix. The pre- and post-expansion stratification isolates the reimbursement change descriptively rather than through a formal interrupted time-series model, so the policy contribution cannot be quantified against a counterfactual, and concurrent shifts in imaging availability, clinician behavior, and coding oversight were not modeled separately. Fifteen annual observations limit the temporal resolution of change-point detection, so the joinpoint years are approximate. The findings apply mainly to East Asian aged populations under universal health coverage. Individual-level microdata with validated case definitions and washout-based case identification would address several of these constraints. The estimates reported here are pooled across sex. A difference between men and women in diagnostic or coding behavior would therefore not be detectable in these data.

## 5 Conclusion

Aging populations are expected to carry a growing burden of degenerative cervical myelopathy. The diagnosis rate recorded in Korean nationwide claims moved in the opposite direction over fifteen years. The age-standardized rate fell by 43.1 percent and the crude patient count by 15.7 percent, while cervical radiculopathy increased. Over the same period cervical surgical volume did not fall, laminoplasty more than doubled, and the operations performed moved toward posterior multi-level decompression. A falling diagnosis rate would at first suggest that the burden of cervical myelopathy is decreasing. The operative record from the same years does not support that interpretation. The authors regard coding, rather than a change in disease occurrence, as the more likely explanation, and some outpatient radiculopathy claims may include mild myelopathic features. Aggregate data cannot settle this question. Individual-level microdata with imaging and clinical severity would be needed to test the interpretation offered here. These diagnostic-prevalence estimates provide a baseline for surveillance of cervical spinal cord disease under universal coverage, and they show that diagnostic and procedural indicators taken from the same claims system need not move in the same direction.

## Data Availability

Publicly available datasets were analyzed in this study. These data can be found at the Health Insurance Review and Assessment Service (HIRA) Open Data Portal (https://opendata.hira.or.kr), which provides annual aggregate counts of primary-diagnosis claims by ICD-10 code (inpatient and outpatient, by sex and 5-year age group), annual counts by procedure fee code, and quarterly medical equipment statistics for 2010–2024, and at the Korean Statistical Information Service (KOSIS), which provides mid-year registered resident population by sex and 5-year age group for 2010–2024 (Table DT_1B040M5 for 2010–2023: https://kosis.kr/statHtml/statHtml.do?orgId=101&tblId=DT_1B040M5; Table DT_1B040M5_1 for 2024: https://kosis.kr/statHtml/statHtml.do?orgId=101&tblId=DT_1B040M5_1). Neither source issues study-level accession numbers. The diagnosis codes, fee codes, calendar years, and stratification variables applied at extraction are specified in Materials and methods. The aggregated input files and the analysis code underlying the tables and figures are available from the corresponding author on request.

## Supplementary material

SUPPLEMENTARY FIGURE S1 Format: TIFF. Title: pre- and post-expansion stratified analysis of age-standardized diagnosis rates. Description: pre- and post-expansion stratified analysis of age-standardized rates around the March 1, 2022 extension of National Health Insurance MRI reimbursement. Top row, degenerative cervical myelopathy: **(A)** 2010–2021, **(B)** 2023–2024. Bottom row, cervical radiculopathy: **(C)** 2010–2021, **(D)** 2023–2024. Year 2022, marked with x at the dotted transition line, is excluded as a transition year. Shaded ribbons and error bars indicate 95 percent confidence intervals. The pre-expansion panels report the AAPC with 95 percent confidence interval and *p* value; the post-expansion panels contain only two annual observations and therefore show the observed rates without a fitted trend line.
